# Supplementary material for: Real world validation of activity recognition algorithm and development of novel behavioral biomarkers of falls in aged control and movement disorder patients
Source: Front Aging Neurosci. 2023 Feb 23;15:1117802. doi: 10.3389/fnagi.2023.1117802 (PMC9995757; doi:10.3389/fnagi.2023.1117802)
Supplement: Supplementary file 1 [file Data_Sheet_1.docx]

**Supplemental Table 1**

| **Num** | **Feature** | **Definition** |
| --- | --- | --- |
|  | Stand | Standing (chest, thigh and shank angles < 20 deg) |
|  | Walk | At least 3 consecutive steps |
|  | Sit | Sitting (thigh angles > 45 deg while chest angle < 20 deg) |
|  | Stand to sit | Transition from standing/walking to sitting |
|  | Sit to stand | Transition from sitting to standing/walking |
|  | Turn | Turning in chest at least 45 deg/s |
|  | Lie down | Chest tilt angle > 45 deg and Thigh tilt angles > 45 deg |
|  | Bend | Bend at least 45 deg while Thigh Angles < 20 deg |
|  | Near-Fall | High forward/backward/lateral acceleration (>10 m/s^2) followed by consecutive balancing steps (>1 step) |
|  | Fall | Free fall, i.e. when the only force acting on body is gravity and the chest acceleration is about g=9.8 m/s^2 |

**Supplemental Table 2**

| **Num** | **Feature** | **Definition** |
| --- | --- | --- |
|  | age | Age of the patient [years] |
|  | height | Height of the patient [centimeters] |
|  | weight | Weight of the patient [kilograms] |
|  | peak_acc | Chest peak acceleration measured in clinical pull tests [meter/second^2] |
|  | mean_acc | Chest average acceleration measured in clinical pull tests [meter/second^2] |
|  | updrs | MDS-Unified Parkinson's Disease Rating Scale measured in clinic |
|  | tot_failure | Total number of failures in clinical pull tests (had to be caught by examiner) |
|  | stp_len | Average step length of the patient in clinical pull tests [meters] |
|  | rxn_time | Average reaction time of the patient in clinical pull tests [seconds] |
|  | rxn_pkAcc_slope | Slope of reaction time vs. peak chest acceleration in clinical pull tests [second^3/ meter] |
|  | rxn_mAcc_slope | Slope of reaction time vs. mean chest acceleration in clinical pull tests [second^3/ meter] |
|  | stpl_pkAcc_slope | Slope of step length vs. peak chest acceleration in clinical pull tests [second^2] |
|  | stpl_mAcc_slope | Slope of step length vs. mean chest acceleration in clinical pull tests [second^2] |
|  | peak_acc_h | Peak chest acceleration measured at home [meter/second^2] |
|  | mean_acc_h | Average chest acceleration measured at home [meter/second^2] |
|  | stp_len_h | Average step length of the patient measured at home [meters] |
|  | Rxn_time_h | Average reaction time of the patient measured at home [seconds] |
|  | rxt_pkAcc_slope_h | Slope of reaction time vs. peak chest acceleration measured at home [second^3/ meter] |
|  | rxn_mAcc_slope_h | Slope of reaction time vs. mean chest acceleration measured at home [second^3/ meter] |
|  | stpl_pkAcc_slope_h | Slope of step length vs. peak chest acceleration measured at home [second^2] |
|  | stpl_mAcc_slope_h | Slope of step length vs. mean chest acceleration measured at home [second^2] |
|  | walk_freq_h | Walking frequency at home (total walking duration / total measurement duration) |
|  | turn_freq_h | Turning frequency at home (total turning duration / total measurement duration) |
|  | bend_freq_h | Bending frequency at home (total bending duration / total measurement duration) |
|  | sit_freq_h | Sitting frequency at home (total sitting duration / total measurement duration) |
|  | lie_freq_h | Lie down frequency at home (total lying down duration / total measurement duration) |
|  | nfall_freq_h | Near-fall frequency at home (total near-falls duration / total measurement duration) |
|  | nfalls_h | Total number of near-falls at home |
|  | totWalk_Min_day | Total duration of walking at home in each day [minutes/day] |
|  | percWalk_day | Percentage of walking at home in each day [%/day] |
|  | totNumABs | Total number of ambulatory bouts *see [18] for details |
|  | meanABdur | Average ambulatory bouts duration [seconds] |
|  | variability | Variability of ambulatory bouts duration [seconds^2] *see [17] for details |
|  | alpha | Alpha for ambulatory bouts *see [17] for details |
|  | totWalk_Min_day_3 | Total duration of walking (counted only walks of more than 3 seconds) at home in each day [minutes/day] *see [18] for details |
|  | percWalk_day_3 | Percentage of walking (counted only walks of more than 3 seconds) at home in each day [%/day] |
|  | totNumABs_3 | Total number of ambulatory bouts (counted only ABs of more than 3 seconds) *see [18] for details |
|  | meanABdur_3 | Average ambulatory bouts duration (counted only ABs of more than 3 seconds) [seconds] *see [18] for details |
|  | variability_3 | Variability of ambulatory bouts duration (counted only ABs of more than 3 seconds) [seconds^2] *see [17] for details |
|  | alpha_3 | Alpha for ambulatory bouts (counted only ABs of more than 3 seconds) *see [17] for details |
|  | totWalk_Min_day_8 | Total duration of walking (counted only walks of more than 8 seconds) at home in each day [minutes/day] *see [18] for details |
|  | percWalk_day_8 | Percentage of walking (counted only walks of more than 8 seconds) at home in each day [%/day] |
|  | totNumABs_8 | Total number of ambulatory bouts (counted only ABs of more than 8 seconds) *see [18] for details |
|  | meanABdur_8 | Average ambulatory bouts duration (counted only ABs of more than 8 seconds) [seconds] *see [18] for details |
|  | variability_8 | Variability of ambulatory bouts duration (counted only ABs of more than 8 seconds) [seconds^2] *see [17] for details |
|  | alpha_8 | Alpha for ambulatory bouts (counted only ABs of more than 8 seconds) *see [17] for details |
|  | fall_freq_per_week | Fall frequency of the patients at home reported in patient diaries in a week [# falls / week] |

**Supplemental Figures**

**
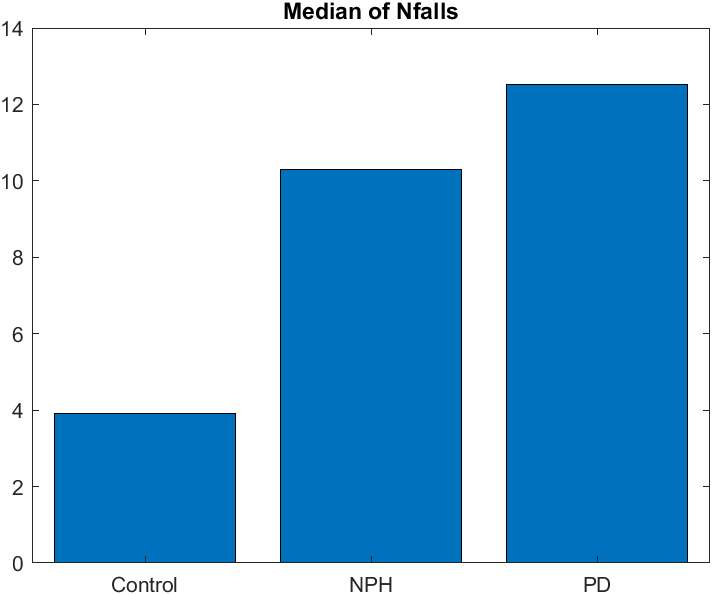
**

Supplemental Fig 1. Comparison of median of number of near-falls per week for PD vs NPH vs C.

Supplemental Fig 2. Comparison of ROC curves for PD vs NPH vs C.
