# Supplementary material for: Real world validation of activity recognition algorithm and development of novel behavioral biomarkers of falls in aged control and movement disorder patients
Source: Front Aging Neurosci. 2023 Feb 23;15:1117802. doi: 10.3389/fnagi.2023.1117802 (PMC9995757; doi:10.3389/fnagi.2023.1117802)
Supplement: Supplementary file 2 [file Table_1.DOCX]

**Tables**

Table 1. Demography of participants

| Condition * | Age [year]  *Mean (SD)* | Female *n, %* | Height [cm]  *Mean (SD)* | Weight [kg]  *Mean (SD)* | UPDRS Pull test  *Mean (SD)* | UPDRS Gait  *Mean (SD)* | Duration of Disease [year]  *Mean (SD)* |
| --- | --- | --- | --- | --- | --- | --- | --- |
| PD (n = 11) | 65 (4.76) | 2, 20% | 181.54 (8.97) | 92.94 (23.63) | 0.56 (0.96) | 0.90 (0.74) | 10.50 (5.41) |
| NPH (n = 8) | 69.8 (8.21) | 1, 11% | 180.72 (3.50) | 102.57 (20.71) | 1.04 (0.56) | 1.85 (0.89) | N/A |
| C (n = 10) | 61.1 (9.97) | 8, 80% | 172.20 (6.83) | 79.75 (27.69) | 0 (0) | 0 (0) | N/A |

* PD = Parkinson’s Disease, NPH = Normal pressure hydrocephalus, C = Control

Table 2. Statistics of each activity in our activity recognition algorithm

| Activity | TP | FP | TN | FN | TPR | TNR | PPV | NPV | ACC |
| --- | --- | --- | --- | --- | --- | --- | --- | --- | --- |
| Stand | 2323 | 54 | 11998 | 14 | 0.994009 | 0.995519 | 0.977282 | 0.998834 | 0.995274 |
| Walk | 5309 | 47 | 9006 | 27 | 0.99494 | 0.994808 | 0.991225 | 0.997011 | 0.994857 |
| Stand to sit | 119 | 0 | 14259 | 11 | 0.915385 | 1 | 1 | 0.999229 | 0.999236 |
| Sit | 300 | 26 | 14045 | 18 | 0.943396 | 0.998152 | 0.920245 | 0.99872 | 0.996942 |
| Sit to stand | 112 | 1 | 14268 | 8 | 0.933333 | 0.99993 | 0.99115 | 0.99944 | 0.999375 |
| Turn | 5414 | 30 | 8898 | 47 | 0.991394 | 0.99664 | 0.994489 | 0.994746 | 0.994649 |
| Lie down | 1 | 3 | 14385 | 0 | 1 | 0.999791 | 0.25 | 1 | 0.999792 |
| Bend | 577 | 5 | 13779 | 28 | 0.953719 | 0.999637 | 0.991409 | 0.997972 | 0.997707 |
| Near-fall | 58 | 5 | 14311 | 15 | 0.794521 | 0.999651 | 0.920635 | 0.998953 | 0.99861 |
| Fall | 2 | 0 | 14387 | 0 | 1 | 1 | 1 | 1 | 1 |

Table 3. Area under the ROC curves for each activity

| Classifier | Standing | Walking | Sit-STANDS | Turning | Bending | Near-Falls |
| --- | --- | --- | --- | --- | --- | --- |
| LoG | 0.85759 | 0.85745 | 0.65548 | 0.69863 | 0.96702 | 0.73180 |
| SVM | 0.99691 | 0.91850 | 0.71383 | 0.78733 | 0.95410 | 0.73401 |
| DT | 0.99999 | 0.99881 | 0.95043 | 0.95940 | 0.98589 | 0.90834 |
| LSTM | 0.99999 | 0.99985 | 0.98920 | 0.99288 | 0.99774 | 0.98253 |

Table 4. Linear regression coefficients for fall frequency prediction

| Feature | Estimate | Standard error | | t-stat | p-value |
| --- | --- | --- | --- | --- | --- |
| intercept | 0.047251 | 0.020211 | 2.337934 | | 0.041485 |
| nfall_freq_h | 0.696391 | 0.188534 | 3.69372 | | 0.004151 |
| totNumABs | 6.82E-08 | 1.62E-06 | 0.042112 | | 0.967239 |
| sit_freq_h | -0.00035 | 0.000178 | -1.99592 | | 0.073889 |
| updrs | -0.01573 | 0.006449 | -2.43948 | | 0.03488 |
| alpha_8 | -0.00391 | 0.001185 | -3.30116 | | 0.007998 |

Table 5. Linear regression model summary for fall frequency prediction

| Feature | SumSq | MeanSq | | F | pValue |
| --- | --- | --- | --- | --- | --- |
| Total | 0.014705 | 0.0009803 |  | |  |
| Model | 0.012228 | 0.0024457 | 9.8737 | | 0.001268 |
| Residual | 0.002477 | 0.0002477 |  | |  |

**Figures**


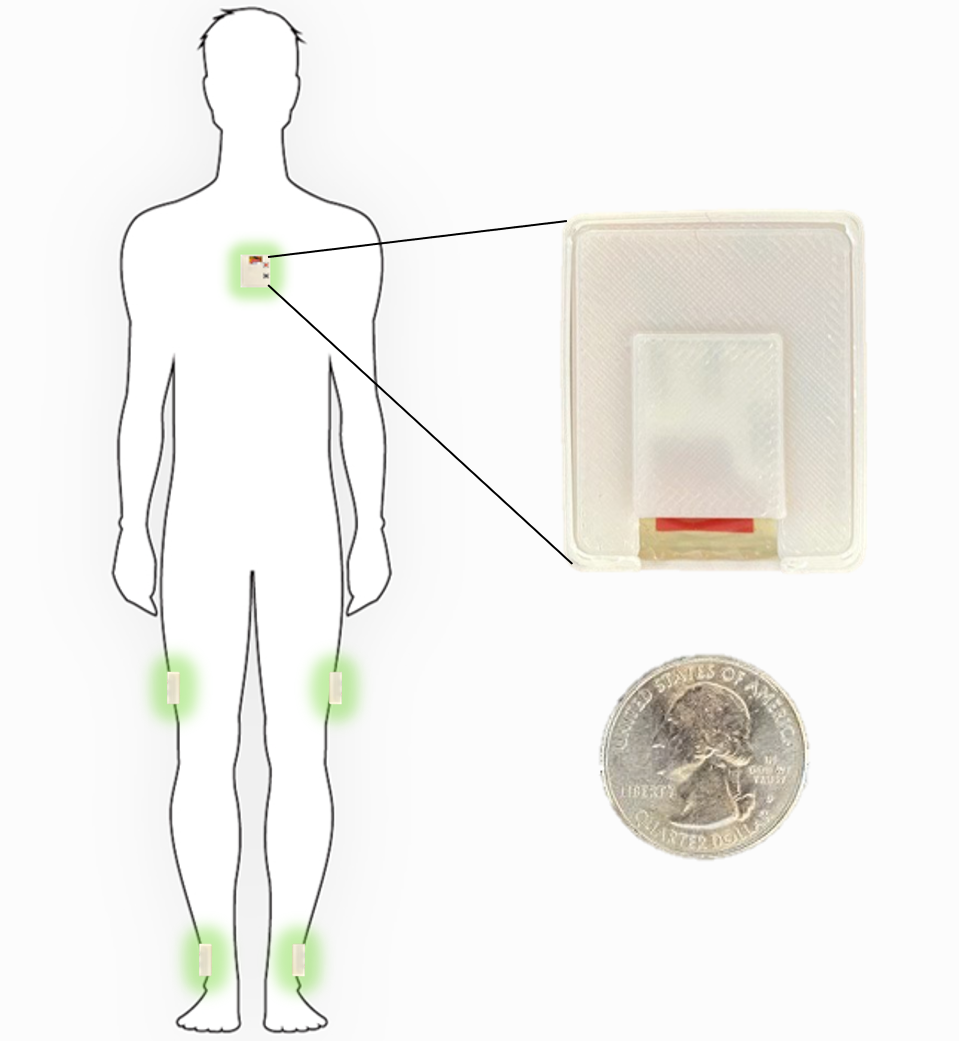


Fig 1. Inertial sensors placement on the body in 5-sensor configuration, one on the chest and one on each lower leg and one on each upper leg.

**
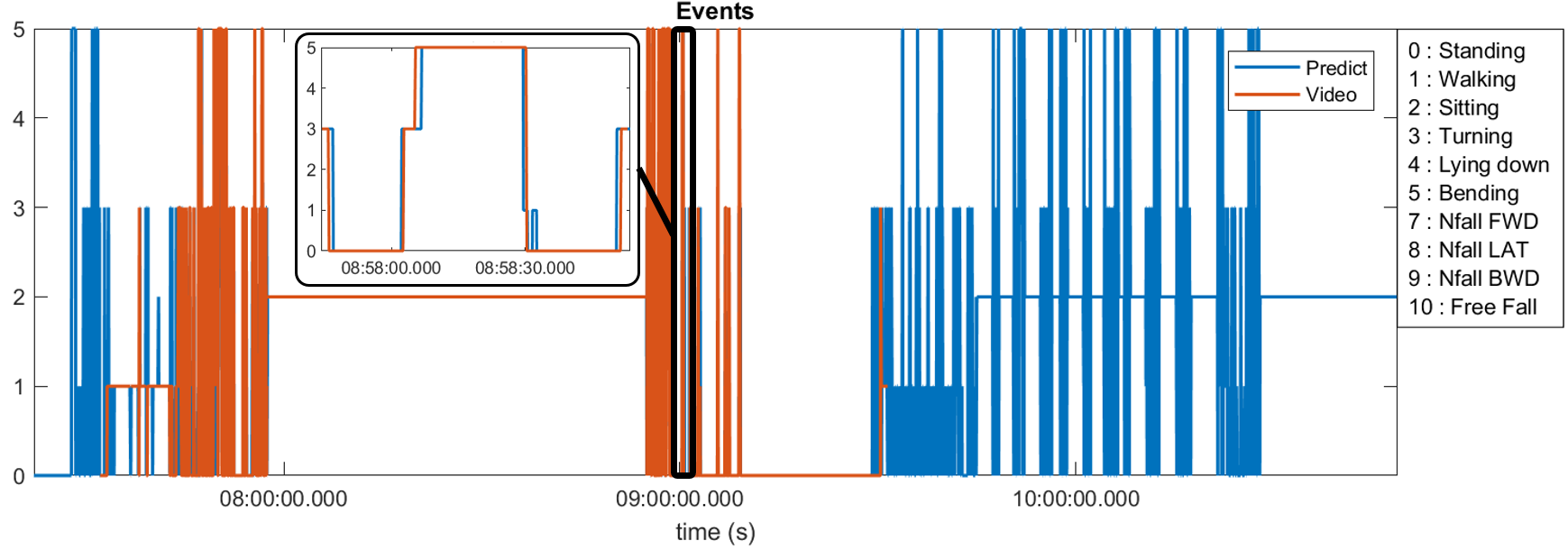
**

Fig 2. Predicted activities compared to the video annotations obtained from a patient at home environment


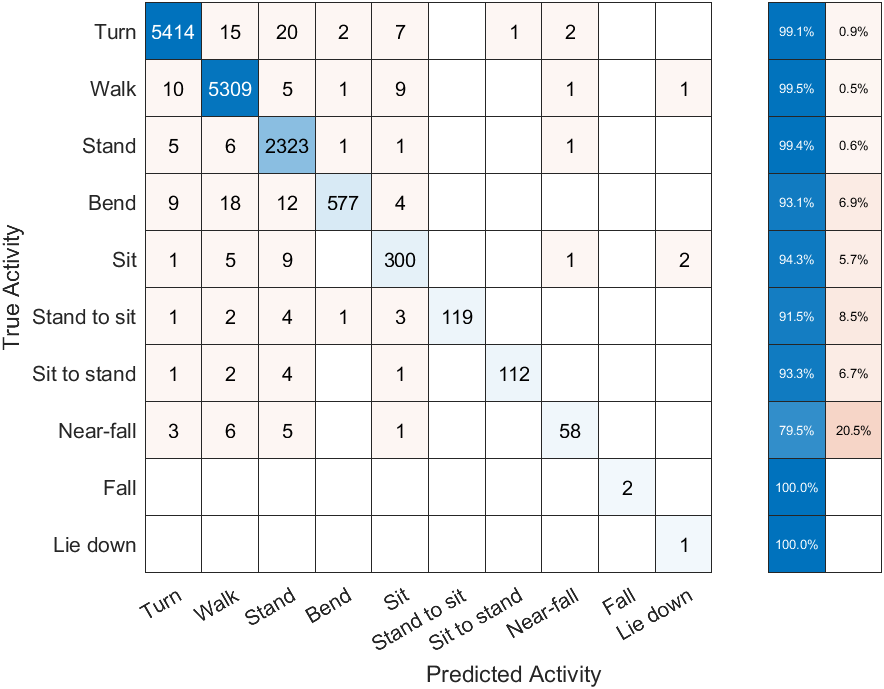


Fig 3. Confusion matrix of activity recognition algorithm results compared to the annotated videos for 6 subjects.

Fig 4. Receiver operating characteristic curves for four binary classifiers plotted separately for each activity.


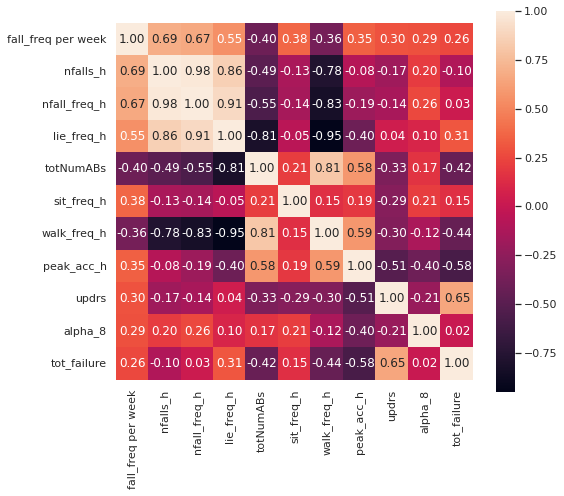


Fig 5. Correlation matrix including the first 10 features with the most correlation with the fall frequency of patients at home.

**Supplemental Table 1**

| **Num** | **Feature** | **Definition** |
| --- | --- | --- |
|  | Stand | Standing (chest, thigh and shank angles < 20 deg) |
|  | Walk | At least 3 consecutive steps |
|  | Sit | Sitting (thigh angles > 45 deg while chest angle < 20 deg) |
|  | Stand to sit | Transition from standing/walking to sitting |
|  | Sit to stand | Transition from sitting to standing/walking |
|  | Turn | Turning in chest at least 45 deg/s |
|  | Lie down | Chest tilt angle > 45 deg and Thigh tilt angles > 45 deg |
|  | Bend | Bend at least 45 deg while Thigh Angles < 20 deg |
|  | Near-Fall | High forward/backward/lateral acceleration (>10 m/s^2) followed by consecutive balancing steps (>1 step) |
|  | Fall | Free fall, i.e. when the only force acting on body is gravity and the chest acceleration is about g=9.8 m/s^2 |

**Supplemental Table 2**

| **Num** | **Feature** | **Definition** |
| --- | --- | --- |
|  | age | Age of the patient [years] |
|  | height | Height of the patient [centimeters] |
|  | weight | Weight of the patient [kilograms] |
|  | peak_acc | Chest peak acceleration measured in clinical pull tests [meter/second^2] |
|  | mean_acc | Chest average acceleration measured in clinical pull tests [meter/second^2] |
|  | updrs | MDS-Unified Parkinson's Disease Rating Scale measured in clinic |
|  | tot_failure | Total number of failures in clinical pull tests (had to be caught by examiner) |
|  | stp_len | Average step length of the patient in clinical pull tests [meters] |
|  | rxn_time | Average reaction time of the patient in clinical pull tests [seconds] |
|  | rxn_pkAcc_slope | Slope of reaction time vs. peak chest acceleration in clinical pull tests [second^3/ meter] |
|  | rxn_mAcc_slope | Slope of reaction time vs. mean chest acceleration in clinical pull tests [second^3/ meter] |
|  | stpl_pkAcc_slope | Slope of step length vs. peak chest acceleration in clinical pull tests [second^2] |
|  | stpl_mAcc_slope | Slope of step length vs. mean chest acceleration in clinical pull tests [second^2] |
|  | peak_acc_h | Peak chest acceleration measured at home [meter/second^2] |
|  | mean_acc_h | Average chest acceleration measured at home [meter/second^2] |
|  | stp_len_h | Average step length of the patient measured at home [meters] |
|  | Rxn_time_h | Average reaction time of the patient measured at home [seconds] |
|  | rxt_pkAcc_slope_h | Slope of reaction time vs. peak chest acceleration measured at home [second^3/ meter] |
|  | rxn_mAcc_slope_h | Slope of reaction time vs. mean chest acceleration measured at home [second^3/ meter] |
|  | stpl_pkAcc_slope_h | Slope of step length vs. peak chest acceleration measured at home [second^2] |
|  | stpl_mAcc_slope_h | Slope of step length vs. mean chest acceleration measured at home [second^2] |
|  | walk_freq_h | Walking frequency at home (total walking duration / total measurement duration) |
|  | turn_freq_h | Turning frequency at home (total turning duration / total measurement duration) |
|  | bend_freq_h | Bending frequency at home (total bending duration / total measurement duration) |
|  | sit_freq_h | Sitting frequency at home (total sitting duration / total measurement duration) |
|  | lie_freq_h | Lie down frequency at home (total lying down duration / total measurement duration) |
|  | nfall_freq_h | Near-fall frequency at home (total near-falls duration / total measurement duration) |
|  | nfalls_h | Total number of near-falls at home |
|  | totWalk_Min_day | Total duration of walking at home in each day [minutes/day] |
|  | percWalk_day | Percentage of walking at home in each day [%/day] |
|  | totNumABs | Total number of ambulatory bouts *see [18] for details |
|  | meanABdur | Average ambulatory bouts duration [seconds] |
|  | variability | Variability of ambulatory bouts duration [seconds^2] *see [17] for details |
|  | alpha | Alpha for ambulatory bouts *see [17] for details |
|  | totWalk_Min_day_3 | Total duration of walking (counted only walks of more than 3 seconds) at home in each day [minutes/day] *see [18] for details |
|  | percWalk_day_3 | Percentage of walking (counted only walks of more than 3 seconds) at home in each day [%/day] |
|  | totNumABs_3 | Total number of ambulatory bouts (counted only ABs of more than 3 seconds) *see [18] for details |
|  | meanABdur_3 | Average ambulatory bouts duration (counted only ABs of more than 3 seconds) [seconds] *see [18] for details |
|  | variability_3 | Variability of ambulatory bouts duration (counted only ABs of more than 3 seconds) [seconds^2] *see [17] for details |
|  | alpha_3 | Alpha for ambulatory bouts (counted only ABs of more than 3 seconds) *see [17] for details |
|  | totWalk_Min_day_8 | Total duration of walking (counted only walks of more than 8 seconds) at home in each day [minutes/day] *see [18] for details |
|  | percWalk_day_8 | Percentage of walking (counted only walks of more than 8 seconds) at home in each day [%/day] |
|  | totNumABs_8 | Total number of ambulatory bouts (counted only ABs of more than 8 seconds) *see [18] for details |
|  | meanABdur_8 | Average ambulatory bouts duration (counted only ABs of more than 8 seconds) [seconds] *see [18] for details |
|  | variability_8 | Variability of ambulatory bouts duration (counted only ABs of more than 8 seconds) [seconds^2] *see [17] for details |
|  | alpha_8 | Alpha for ambulatory bouts (counted only ABs of more than 8 seconds) *see [17] for details |
|  | fall_freq_per_week | Fall frequency of the patients at home reported in patient diaries in a week [# falls / week] |

**Supplemental Figures**

**
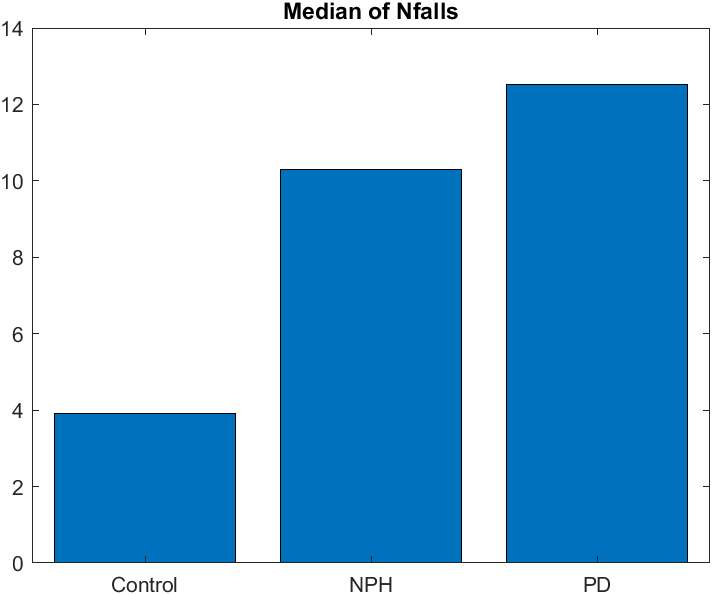
**

Supplemental Fig 1. Comparison of median of number of near-falls per week for PD vs NPH vs C.

Supplemental Fig 2. Comparison of ROC curves for PD vs NPH vs C.
